# CLC-2 single nucleotide polymorphisms (SNPs) as potential modifiers of cystic fibrosis disease severity

**DOI:** 10.1186/1471-2350-5-26

**Published:** 2004-10-26

**Authors:** Carol J Blaisdell, Timothy D Howard, Augustus Stern, Penelope Bamford, Eugene R Bleecker, O Colin Stine

**Affiliations:** 1 Department of Pediatrics, School of Medicine, University of Maryland, Bressler 10–019, 655 W. Baltimore St., Baltimore, Maryland, 21201 USA; 2 Center for Human Genomics, Wake Forest University School of Medicine, Medical Center Blvd., Winston-Salem, North Carolina, 27157 USA; 3 School of Medicine, University of Maryland, Howard Hall 324, Baltimore, Maryland, USA; 4 Department of Genetics, School of Medicine, University of Maryland, Howard Hall 596, 660 W. Redwood St., Baltimore, Maryland, USA

## Abstract

**Background:**

Cystic fibrosis (CF) lung disease manifest by impaired chloride secretion leads to eventual respiratory failure. Candidate genes that may modify CF lung disease severity include alternative chloride channels. The objectives of this study are to identify single nucleotide polymorphisms (SNPs) in the airway epithelial chloride channel, CLC-2, and correlate these polymorphisms with CF lung disease.

**Methods:**

The CLC-2 promoter, intron 1 and exon 20 were examined for SNPs in adult CF dF508/dF508 homozygotes with mild and severe lung disease (forced expiratory volume at one second (FEV1) > 70% and < 40%).

**Results:**

PCR amplification of genomic CLC-2 and sequence analysis revealed 1 polymorphism in the hClC -2 promoter, 4 in intron 1, and none in exon 20. Fisher's analysis within this data set, did not demonstrate a significant relationship between the severity of lung disease and SNPs in the CLC-2 gene.

**Conclusions:**

CLC-2 is not a key modifier gene of CF lung phenotype. Further studies evaluating other phenotypes associated with CF may be useful in the future to assess the ability of CLC-2 to modify CF disease severity.

## Background

Although greater than 1000 mutations of the CF gene product, CFTR are known, none of these can be used to make predictions about the occurrence of common complications, the severity, or course of pulmonary disease. The identification of a gene, which modifies the phenotypic expression of CF would be very important for understanding this complex disease. Because CF is a disease of chloride transport in respiratory epithelia, alternative chloride channels present in the airway may be able to partially compensate for the CF defect.

CLC-2 is one candidate alternative chloride channel in respiratory epithelia. Localization to the luminal surface of the airway and perinatal downregulation of CLC-2 in mammalian lung suggests a role in lung morphogenesis [1,2]. Persistent expression of CLC-2 mRNA and protein in tissues unaffected in CF suggests that CLC-2 may compensate for defects in CFTR expression [1]. CLC-2 has the capacity to conduct chloride in mature respiratory epithelia [3,4]. The rat CLC-2 promoter has SP-1 domains that are important for gene regulation [5]. A splice variant of CLC-2 skipping exon 20 occurs in rat lung, suggesting that alternative splicing may have functional significance in this tissue [6]. Because investigation of human CLC-2 genomic structure would be important for studies of gene regulation, we sought to identify single nucleotide polymorphisms in potential regulatory domains of human CLC-2. Genomic DNA was isolated from CF adults with severe and mild obstructive lung disease in order to determine if SNPs segregate with CF lung phenotype.

## Methods

### CLC-2 protein expression in CF nasal polyps

Nasal polyps from CF patients were obtained at the time of elective surgery from 1989 to 1996. Genotypes of CF mutations for each patient was available, but not clinical status, according to approval by the Johns Hopkins Medical Institution Review Board. At harvest, the tissue was washed 3 times in HBSS, and incubated at 4°C overnight in Protease XIV (Sigma). Epithelial cells were isolated by gentle agitation and filtered through a 70-μm nylon cell strainer (Becton Dickinson; Franklin Lakes, NJ). Cells were grown on 1% collagen coated 35 mm dishes for 1 week. Cell lysates were prepared using 2% sodium dodecyl sulfate (SDS) at 65°C and a cell scraper. Equivalent amounts of total protein from primary CF nasal polyp cultured cell lysates were loaded onto an SDS-polyacrylamide gel electrophoresis (PAGE) system, electrophoresed and transferred to a nitrocellulose membrane. CLC-2 protein levels were detected using the polyclonal chicken anti-CLC-2 antibody and the enhanced chemiluminescent reaction as previously described [2].

### Population studied for CLC-2 polymorphisms

Variable expression of CLC-2 protein in nasal cell lysates (see Results) suggested that CLC-2 is differentially expressed in adults and that examination of human CLC-2 genomic structure would be important to investigate this differential expression. Identification of volunteers for nasal epithelial cell culture was not permitted with the original IRB consent process. Therefore, a cohort of CF patients was recruited for polymorphism analysis. A review of the Johns Hopkins Medical Institution CF center database was conducted in 1998 to identify patients that had reached adulthood (age > 18 years), homozygous for the most common CF genotype delF508, so that the affect of various CFTR genotypes would not affect the investigation of CLC-2 polymorphisms. Status of obstructive lung disease was defined using most recent pulmonary function studies. Those patients with spirometry FEV1 ≤ 40% predicted were classified as severe, those with spirometry FEV1 ≥ 70% predicted as mild in order to classify 2 severity levels of CF lung disease. Of 74 eligible subjects (age > 18 years, del F508 homozygous), 43 had FEV1 ≥ 70%, 9 had FEV1 = 41–69%, and 22 had FEV1 ≤ 40%; 31 were recruited during routine visits to the CF center from June 1998 to January 2000. With informed consent, participants provided blood samples for genomic DNA isolation. This study was approved by the Institutional Review Board at Johns Hopkins Medical Institution. DNA was isolated from lymphocytes using standard procedures.

### Identification of CLC-2 polymorphisms

The genomic structure of rat CLC-2 has been previously published [5,6]and has important sites for gene regulation. The human CLC-2 genomic sequence, however, was largely unknown at the start of this study. Promotors are an important site to examine for SNPs, which might affect regulation of a gene. The first intron of a gene also can function as an important regulatory domain. Because the rat lung has a splice variant that deletes exon 20 [6] due to an unusually high CT content in the upstream intron 19 and a rare AAG acceptor site, this region was also examined for polymorphisms.

Primer pairs were thus chosen from rat [accession gi|4406230] and human CLC-2 sequence [accession S7770] to amplify the promoter, intron 1 and exon 20 from adult CF subjects homozygous for delF508. Sequencing of the human CLC-2 promoter initially from one human genomic sample was performed by polymerase chain reaction using the 5'-flanking region of rat hpolE1 (dTCC GGG TCA ATA TCC TTC ACA TCG), which is approximately 2000 base pairs upstream from the rat CLC-2 coding sequence [5] and the 3'-hCLC-2 promoter primer (dCGC CCG TGG CTC CAT CCC TTC), which corresponds to sequence from the N-terminus of the hCLC-2 coding region [accession S7770 [7]]. PCR amplification was performed using the MasterAmp™ PCR Optimization Kit buffer J (Epicentre Technologies, Madison, WI) due to the high GC rich content of this region in the rat [5]. The amplified product was cloned into the TA cloning Vector (Invitrogen), plasmid DNA grown in *E. coli*, and DNA isolated using the Mini-prep kit (Qiagen, Valencia, CA) according to the manufacturer's instructions. The sequence of this ~2000 bp product yielded genomic DNA for design of primer pairs that would yield overlapping PCR-amplified DNA fragments of the promoter.

Sequencing and identification of human CLC-2 promoter polymorphisms in 15 CF patients with severe obstructive lung disease (FEV1 ≤ 40% predicted) and 16 CF patients with mild disease (FEV1 ≥ 70% predicted) were performed by polymerase chain reaction using overlapping primers designed from the initial hCLC-2 clone. The only product that yielded a SNP was amplified using primers 15F dGTC CCA GGA GTA GAC TTC C and 16R dCAC TGC CCT CTG GCC TC providing a 760 base pair product, using cycling conditions of 94°C 6 mins, 35 cycles of 94°C 30 s, 59°C 30 s, 72°C 30 s and 72°C 6 min. A nested reaction with 20 uM primers 17F dTCC CCT CCG GCC TAC CCC TTC CGG T and 18R dGGA AGG ATT CGG AGA GGG TTG GGG C amplified both a 150 and 300 bp product using Epicentre MasterAmp™ buffer J (Madison, WI) with cycling conditions 94°C 6 mins, 35 cycles of 94°C 30 s, 64°C 30 s, 74°C 30 s and 74°C 6 min.

Because regulation of a gene may occur also through its first intron we amplified this region from all subjects using primers 1F' dCGC TGC AGC ACG AGC AGA C and 1R' dCCC AAG GTC CTG AGT GTA CC, which yielded a product 2273 bp product. Cycling conditions were 95°C 6 mins, 35 cycles of 95°C 30 s, 63°C 30 s, 72°C 3 minutes and 72°C 6 min. Finally, because exon 20 is alternatively spliced in rat lung [6] we examined whether or not SNPs existed in this region including parts of exon 19 and 21 and the intervening introns using primers 20F dGCC TCT TCT GTG GCA GTC C and 20R dCTT CAG GGC TCA TCT CGC C using PCR amplification conditions of 92°C 6 mins, 30 cycles of 92°C 30 s, 55°C 30 s, 72°C 30 s and 72°C 6 min These amplify a 481 bp fragment covering the 3' end of E19 to 5' end of E21.

With PCR amplification of all 31 genomic CF samples using primers listed in Table 1, the presence of amplified products was confirmed on agarose gels. Amplified DNA and primers were separated using Millipore filters. The purified PCR products were sequenced in both directions using the same primers used for amplification and Big Dye cycle sequencing kit (ver. 2 or 3.1, ABI) in accordance with the manufacturer's instructions. The fluorescently labeled products were separated and detected using either an ABI 377 or 3700 or 3730xl Automatic Sequencer (ABI). The trace files were read using Phred [8,9] and Phrap [10]. Each potential polymorphism was confirmed by visual inspection.

**Table 1:** Primers used to amplify CLC-2 polymorphisms

**Primer**	**oligomer**	**expected size (bp)**
rat hpolE1	dTCC GGG TCA ATA TCC TTC ACA TCG	
		2128
hClC-2 promoter	dCGC CCG TGG CTC CAT CCC TTC	
15F	dGTC CCA GGA GTA GAC TTC C	
		760 bp
16R	dCAC TGC CCT CTG GCC TC	
17F	dTCC CCT CCG GCC TAC CCC TTC CGG T	
		147 + 300 bp
18R	dGGA AGG ATT CGG AGA GGG TTG GGG C	
Intron 1F	dCGC TGC AGC ACG AGC AGA C	
		2273
Intron 1R	dCCC AAG GTC CTG AGT GTA CC	
Exon 20F	dGCC TCT TCT GTG GCA GTC C	
		481
Exon 20R	dCTT CAG GGC TCA TCT CGC C	

## Results

### Expression of CLC-2 protein in CF nasal cells

**Figure 1:**
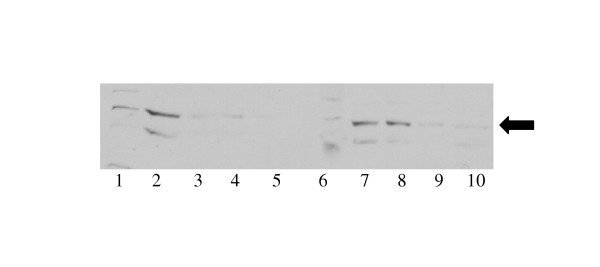
ClC-2 expression by Western blot of nasal polyp lysates from CF adults with the following genotypes: Lanes 1,3,6 dF508/dF508; Lane 2: dF508/d559T; Lane 4: unknown; Lane 5: S549N/R553X; Lane 7,9: dF508/unknown; Lane 8: F508/W1282X.; Lane 10, IB3-1 cell line, genotype F508/W1282X. Arrow identifies CLC-2 bands.

CLC-2 protein is nearly undetectable in postnatal rat lung [2], however we hypothesized that postnatal expression of CLC-2 in CF individuals might confer a protective advantage for the respiratory epithelium of CF individuals. We examined human CLC-2 protein expression using lysates from primary nasal cells obtained from elective polypectomy of CF patients with a variety of CFTR mutations. Similar amounts of total protein from nasal lysates electrophoresed on an SDS-PAGE system had variable amounts of CLC-2 protein detected [figure 1]. High levels of CLC-2 protein were expressed in some lysates, but CLC-2 protein was nearly undetectable in others suggesting that CLC-2 expression is variably regulated in humans. CFTR genetic mutation information was available for these patients and did not correlate with levels of CLC-2 protein expressed [figure 1]. In addition, the expression of ClC-2 protein was diminished in transformed bronchial epithelial IB3-1 cells [11] (lane 10, figure 1), which were derived from primary nasal epithelial cells of a subject with delF508/ W1282X (lane 8, figure 1). While data about the genetic mutations of the CFTR were available on these patients, information about their clinical status was not according to an agreement with the Johns Hopkins Institutional Review Board.

### Single nucleotide polymorphisms in CLC-2

In order to minimize the confounding of genotype, race and age, all individuals were homozygous for delF508 mutation of CFTR, Caucasian, and over 17 years old. FEV1% determined 2 cohorts, one with mild CF lung disease with average FEV1% of 77.4 ± 3.18 SEM (Table 2, n = 16, 9 male). The group with severe lung disease had an average FEV1% of 35.6 ± 3.13 SEM (n = 15, 9 male). The mean age of the mild and severe groups was not significantly different (22.6 ± 1.37 years vs. 24.7 ± 1.56 years mean ± SEM). Because CLC-2 expression could be regulated through the promoter, for each patient's DNA, we amplified the CLC-2 promoter, primers that produced overlapping sequences that were examined for SNPs. In addition, intron1 and exon 20 were investigated for SNPs because of their potential role in CLC-2 expression.

**Table 2:** Demographics of study subjects.

	**FEV1**	**Gender**	**Age (years) (± SEM)**
**Severe**	35.6 (3.13)	9 M / 6 F	24.7 (1.56)
**Mild**	77.4 (3.18)	9 M / 7 F	22.6 (1.37)

### Promotor

**Figure 2:**
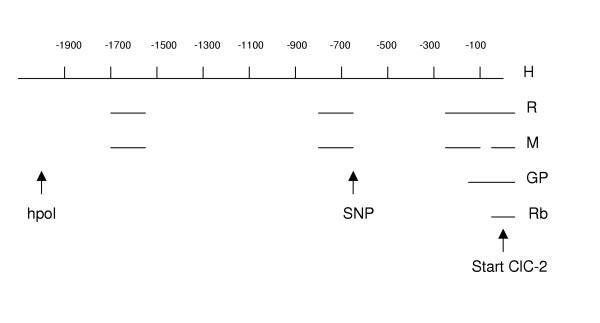
Diagram of alignment of human CLC-2 promoter and mammalian homologues (H = human, R = rat, M = mouse, GP = guinea pig, and Rb = rabbit. CLC-2 translation initiation site in all 5 species is denoted by "start". One single nucleotide polymorphism (SNP) is present at nt -693 (human). Hpol is a polymerase whose gene product is on the complementary strand, upstream from the CLC-2 promoter.

PCR amplified a 2128 bp promotor product confirmed by agarose gel. Sequence comparison revealed that bp 21 to 2128 of the amplified sequences was compatible with bp 317320 to 319427 of ref|NT_0292533|Hs_29412 and that there were no differences between the two sequences. Examination of these products determined that the upstream region was RPB8 exons 1–3 of the human gene polr2H (gi|8052522|) as expected from the rat genomic structure [5]. Human CLC-2 promoter is 69% GC rich and contains 4 GC boxes in the 225 bp upstream from the ATG start site (sequence to submit to GenBank). This area is very similar to rat ClC-2 promotor, where binding of transcription factors Sp1 and Sp3 occurs [5]. Human CLC-2 promotor sequence is very conserved with as much as 82% sequence identity with rat (gi 4406230) and 77% with mouse (gi 28494743). Guinea pig genomic sequence (gi 5001715) aligns with approximately 100 bp of the terminal end of human CLC-2 promoter and rabbit (gi 642465) only with 19 bp upstream of the coding sequence (Figure 2a and 2b). One G/A polymorphism was identified in the 5' upstream sequence of human CLC-2. This SNP is -693 relative to the ATG start site of hCLC-2 (figure 2b, asterisk, genbank S7770), and has not previously been identified. The -693 G/A polymorphism is a putative AP-2 binding site, predicted by TESS and MATINSPECTOR [12,13], which may affect regulation of the gene.

There were five subjects with severe CF lung disease (FEV1 < 40%), who had the genotype A/G, whereas eleven had G/G at position -693 (Table 3). Of the individuals with mild CF lung disease (FEV1 > 70%), 6 had A/G and 9 had G/G. By Fisher's test analysis there was no difference in the frequency of the promotor polymorphism between the severe and mild groups (p = 0.72).

**Table 3:** Promotor & Intron 1 hClC-2 polymorphisms

	Promotor		Intron 1		
	-693	358	427	1089	1909
FEV1 <40	AG (5)	GG (13)	AA (13)	TT (9)	GG (15)
	GG (11)	GC (2)	AG (2)	CT (6)	GC (0)
FEV1 >70	AG (6)	GG (11)	AA (11)	TT (6)	GG (12)
	GG (9)	GC (3)	AG (3)	CT (10)	GC (2)
P-value	0.72	0.32	0.32	0.21	0.22

### Intron 1

**Figure 3:**
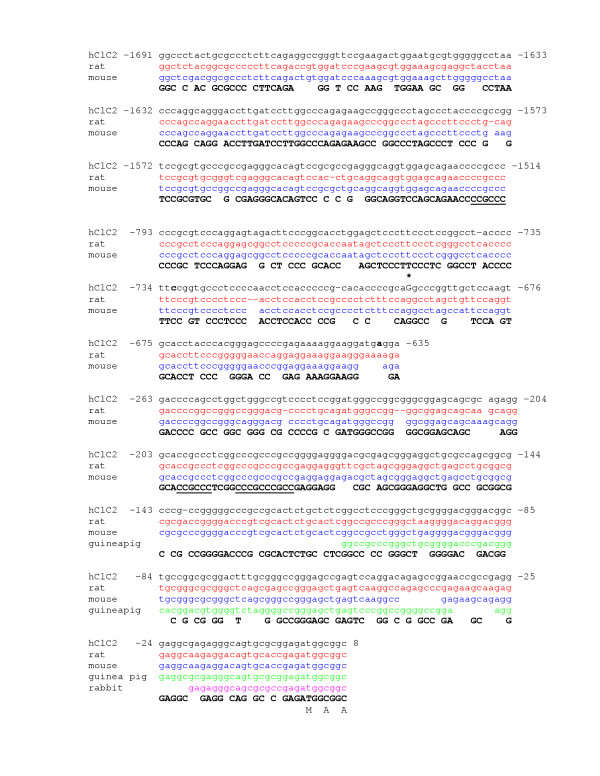
Sequence alignment of human, rat, mouse, guinea pig, and rabbit CLC-2 promoter. Site of human SNP at position -693 shown with asterisk. Conserved GC boxes underlined.

The first intron of human CLC-2 was amplified and the 2273 bp product confirmed by gel electrophoresis. This sequence correlates with bp 319453 to 321725 of ref|NT_0292533|Hs_29412. Human CLC-2 intron 1 has regions with as much as 74% sequence identity with rat (gi 2873366) and 85% with mouse (gi28494743) (Figure 3a and 3b). Examination of 31 human CF samples revealed four SNPs: 358 G/C, 427 A/G, 1089 T/C and 1909 G/C (Figure 3a). There is complete linkage disequilibrium between SNP 358 and 427. Two CF subjects with severe lung disease (FEV1 < 40%) had 358 G/C, 2 had 427 A/G, and 6 had 1089 C/T, 0 had 1909 G/C (Table 3). Of the mild subjects (FEV1 > 70%), 3 of 14 had 358 G/C, 3 of 14 had 427 A/G, 10 of 16 had 1089 C/T, and 2 of 14 had 1909 G/C. By Fisher's test analysis there was no difference in the frequency of any one of the intron 1 polymorphisms between the severe and mild groups (Table 3, p = 0.32, 0.32, 0.21, and 0.22 for SNPs 358, 427, 1082 and 1902 respectively).

### Exon 20

Primers used to examine the potential exon 20 splice variant region in hCLC-2 amplified a 481 bp fragment that correlates with bp 328123 to 328556 of human genomic sequence NT_0292533 and 2446 to 2617 of hCLC-2 cDNA (accession S7770). There were no SNPs identified using all 31 patient samples.

## Conclusions

With an autosomal recessive pattern of inheritance, CF was long considered a monogenic disease with 1 mutant allele inherited from each parent. While CF neonatal screening is offered in several states of the U.S., counseling of families has been difficult, because CF genotyping does not easily predict onset and severity of pulmonary complications [14]. Strategies to identify modifier genes for the CF phenotype are important for defining disease prognosis and developing new strategies to prevent progression of the disease.

There are several chloride conductances, which have been characterized in the mammalian lung: the cAMP-dependent cystic fibrosis transmembrane conductance regulator (CFTR) [15], the Ca^++^-dependent chloride channel (CaCC) [16-18], the outwardly rectifying chloride channel (ORCC) [19], the purinergic receptor-mediated chloride channel [20,21], and the voltage- and volume-regulated, ClC family of chloride channels [3,22-24].

One or more of the chloride channels present in the respiratory epithelium may be able to partially compensate for defects in another. For example, there was no lung pathology in the first CF knock-out mouse models, where there is enhanced activity of a Ca^++^-dependent chloride channel [25-27], however lung disease is present when alternative chloride channels are absent [26]. The CF mouse, however, develops severe intestinal disease leading to premature death, which has been attributed to inadequate secretion via alternative chloride channels. Ca^++^-dependent chloride conductance is low in the intestine of the CF knock-out mouse. To take advantage of alternative chloride channels in the lung, UTP analogues have been used to stimulate chloride secretion in CF individuals via the purinergic receptor-mediated chloride channels [20,21].

One member of the ClC family of chloride channels may also be an alternative chloride conductance in the airway epithelium. We have demonstrated that CLC-2 mRNA and protein are abundantly expressed in the fetal lung [1,2] and that acidic pH can activate chloride secretion [3,22]. CLC-2 mRNA and protein are much higher in brain and kidney compared to tissues that are more severely affected by defective CFTR (lung, intestine, liver) [1] suggesting that CLC-2 expression may protect against disease manifestations in certain tissues. CLC-2 immunolocalizes to the apical surface of the respiratory epithelium [2,22], consistent with the potential to function as a chloride channel in a secretory organ. In this study, we have shown that several CF subjects do express CLC-2 protein as adults (figure 1), unlike in rats [2]. In single channel recordings, overexpression of CLC-2 in a CF bronchial epithelial cell line demonstrated that chloride secretion can be enhanced [3]. While the CLC-2 knock-out mouse has degeneration of the retina and testes [28], loss of CLC-2 function has not been associated with lung disease. To date overexpression of CLC-2 has not been described in an animal model to determine if this channel can be upregulated and serve as a potential therapeutic target for CF.

In this study of 31 CF subjects, we identified 5 single nucleotide polymorphisms that have not previously been described for human CLC-2. One of these is -693 relative to the ATG start site of hCLC-2 (Genbank S7770). The -693 G/A polymorphism is a putative AP-2 binding site, predicted by TESS and MATINSPECTOR [12,13] and may be important for regulation of the gene. This polymorphism was no more frequent in the CF subjects with mild lung disease compared with the subjects with severe lung disease.

In the rat, SP-1 sites are important for gene regulation [5,29,30]. ClC-2 expression in the lung is developmentally downregulated at birth [2] and is dependent on Sp binding to GC boxes in the ClC-2 promotor [5]. These GC boxes are highly conserved in human and rat, suggesting they are important sites for gene regulation. Phosphorylation of Sp-1 decreases its DNA binding activity and coincides with the downregulation of CLC-2 expression [5]. SNPs in the conserved GC boxes were not identified in the subjects of this study.

We also identified 4 polymorphisms in hCLC-2 intron1. These did not appear more or less frequently in the mild CF subjects. Two of the polymorphisms were in complete linkage disequilibrium. The polymorphisms were not identified in areas that were highly conserved in rat or mouse. While splice variants of exons may be affected by intron/exon boundaries, we did not find any polymorphisms in the region of the rat exon 20 splice variant [6].

These findings suggest that CLC-2 is not regulated differently at the genomic level in relatively healthy CF adults. Lack of an association in this study does not exclude the possibility that CLC-2 plays a role in modifying the CF phenotype as might be suggested by the variability of CLC-2 protein expression in primary respiratory epithelial cells from CF subjects in this study. Although we were limited by inadequate power with a small sample size and because phenotypic contrast was low, our data suggest that gene regulation of CLC-2 in relation to polymorphisms in regulatory domains does not play a major role in protection against CF lung disease. Studies which rely on recruitment of small numbers of patients have been shown to detect a difference when a strong relationship is present [31]. Another limitation may be in the selection of FEV1 at a single point in time, rather than using rate of decline of FEV1. Other studies of CF modifier genes have similarly found difficulty in confirming a candidate gene, which also relied on FEV1 at one time point. In addition, the effect on lung phenotype may occur at an earlier stage of CF lung disease, and examination of adults only as in this study may have limited our ability to detect a difference. The polymorphisms identified in this report should facilitate further investigation of CLC-2 regulation. While we did examine subjects with the same CF genotype (namely delF508 homozygous), measures of ion transport (sweat chloride, nasal potential difference), time to colonization with Pseudomonas, and frequency of pneumonia, should be taken into account in future studies.

The identification of candidate genes, which may modify CF lung disease is important so that new therapies may be developed. Multi drug resistant genes have recently been identified that provide some "protection" to the CF lung phenotype [32]. Ion transport dysfunction of CFTR and the channels it regulates, however, may not be the only determinant of disease severity. Many have suggested that inflammatory mechanisms may also impact disease progression and survival in CF individuals [33]. Other classes of candidate genes possibly related to CF phenotype include tumor necrosis factor alpha (TNF-α), nitric oxide synthase (NOS), alpha 1-antitrypsin, mannose-binding lectin [34], and other ion channels such as the basolateral K+ channels [17,35-38].

Lastly, gene expression and function may be independent of genomic polymorphisms, as suggested by our data demonstrating variable expression of hClC-2 protein in CF nasal polyps and must also be considered as a mechanism whereby, CLC-2 could alter the course of CF disease. Haug et al. recently identified mutations in the CLC-2 coding region that are associated with idiopathic generalized seizures in humans [39]. No lung disease was reported from loss of function of CLC-2, so presumably CLC-2 is not critical for the function of mature respiratory epithelium when CFTR is present. A ClC-2 knock-out mouse shows severe degeneration of the retina and testes, but no evident lung disease [28,40]. While there has been no report of ClC-2 lung abnormality in these mice, they do not replicate the human seizure disorder and mouse models do not exclude the possibility of a role in airway epithelial ion transport. For example, initial studies of CF knock out mice also suggested no discernible lung disease that mimics CF in humans [41,42]. The activation of CLC-2 currents by acidic pH, suggests that alterations of key regulatory domains of the channel may affect function. There is disagreement about whether or not a specific region of the N-terminus of CLC-2 is the sensor for acid and voltage regulation [43-45].

This study provides important information about the human CLCN2 genomic organization. Several polymorphisms of key regulatory domains of CLCN2 were identified in a cohort of subjects with cystic fibrosis, who carry the same CF genotype. While we have found no significant association of CLC-2 polymorphisms with FEV1 % predicted in adulthood, further study of potential polymorphisms in CF subjects at an earlier age and investigation of potential mutations in the coding region of CLC-2 that would lead to enhanced transepithelial chloride transport would be necessary to determine if CLC-2 can modify CF.

## Competing interests

The author(s) declare that they have no competing interests.

## Authors' contributions

CB provided overall study design, analysis, and drafted the manuscript. TH designed sequencing methods and analyzed alignment. AS and PB conducted experiments. EB contributed to the design of the study and OS designed sequencing methods and provided analysis including statistics.

All authors read and approved the final manuscript.

**Figure 4:**
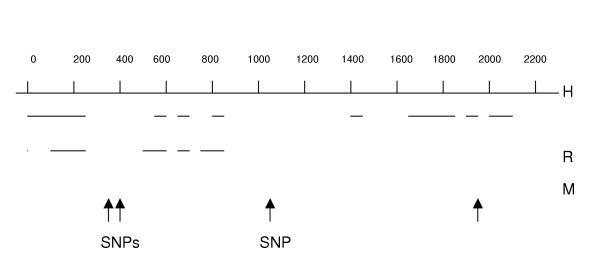
Diagram of alignment of human CLC-2 intron1 and mammalian homologues (H = human, R = rat, and M = mouse. Four single nucleotide polymorphisms (SNP) are present at nt 358, 427, 1089, and 1909 (human).

**Figure 5:**
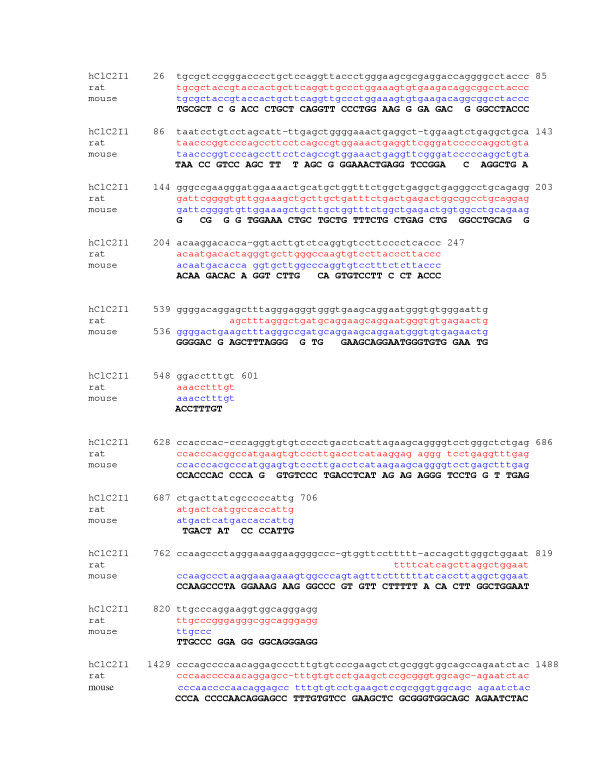
Sequence alignment of human, rat, and mouse CLC-2 promoter (nt 26-1488).

**Figure 6:**
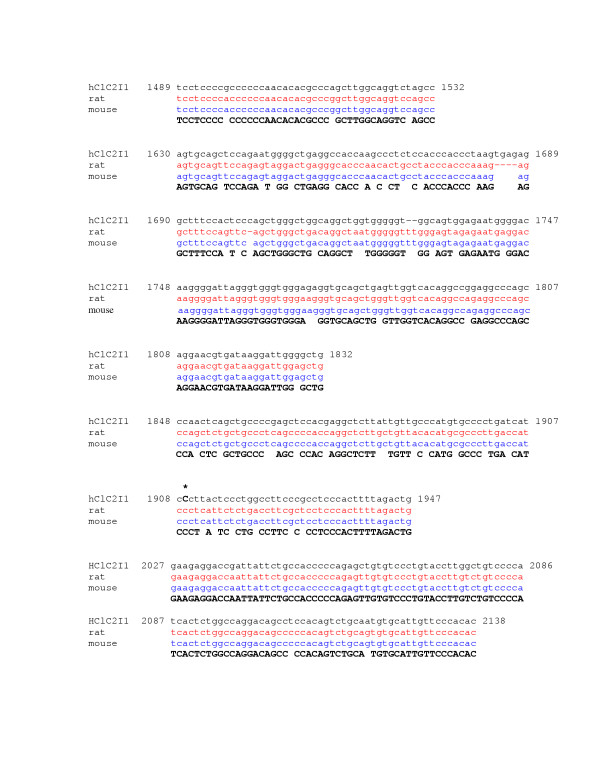
Sequence alignment of human, rat, and mouse CLC-2 promoter (nt 1489-2138). Site of human SNP at position 1909 shown with asterisk.

## Pre-publication history

The pre-publication history for this paper can be accessed here:


